# Is there a role for cementless primary stem in hip arthroplasty for early or late fixation failures of intertrochanteric fractures?

**DOI:** 10.1186/s12891-022-05223-x

**Published:** 2022-03-18

**Authors:** Hsuan-Hsiao Ma, Te-Feng Arthur Chou, Shang-Wen Tsai, Cheng-Fong Chen, Po-Kuei Wu, Wei-Ming Chen

**Affiliations:** 1 Department of Orthopaedics and Traumatology, Taipei Veterans General Hospital, No. 201, Sec 2, Shi-Pai Road, Taipei, 112 Taiwan; 2 Department of Orthopaedics, School of Medicine, National Yang Ming Chiao Tung University, Taipei, Taiwan

**Keywords:** Cementless, Complication, Failure, Intertrochanteric fracture, Stem, Total hip arthroplasty

## Abstract

**Background:**

The choice of femur stems during the hip arthroplasty procedures for patients with treatment failure of intertrochanteric fractures (ITF) remains controversial. We aimed to compare the surgical complication and reoperation rates between cementless primary and revision stems in the early (≤3 months) and late (> 3 months) fixation failures of ITF.

**Methods:**

This was a retrospective, cohort study conducted in a single, tertiary referral hospital of Taipei, Taiwan. We included hip arthroplasty procedures for failed ITF using cementless primary or revision stems. There were 40 and 35 patients who had early and late fixation failure of ITF, respectively. The patient demographics, time to fixation failure, surgical complications and medical complications were recorded for analysis.

**Results:**

We included 75 patients that underwent hip arthroplasty procedure for failed ITF using cementless primary (*n* = 38) or revision (*n* = 37) stems. The mean age was 79.3 years and 56% of the patients were female. In the early fixation failure group, the complication rate was similar between the primary and revision stems (44% vs. 29%, *p* = 0.343). However, there was a trend toward a higher reoperation rate (31% vs. 8%, *p* = 0.061) of using the primary stem, compared with the revision stem. In the late fixation failure group, the rate of complication and reoperation was similar between the two stem types.

**Conclusion:**

For early fixation failures of ITFs, we caution against the use of cementless primary stems due to a trend towards an increased risk of reoperations compared to the use of cementless revision stems. However, in late fixation failures of ITFs, there is a role for cementless primary stems.

**Level of evidence:**

III, retrospective cohort study.

**Supplementary Information:**

The online version contains supplementary material available at 10.1186/s12891-022-05223-x.

## Introduction

Conversion hip arthroplasty is the mainstay of treatment for failed intertrochanteric fractures (ITF) [1–3]. Several failure modes after internal fixation of an ITF have been discussed, including the cut-out of lag screws, hardware breakage or failure, avascular necrosis of femoral head, and secondary hip osteoarthritis [4, 5]. The distorted soft tissue and bony anatomy, osteoporotic bones, critical bone defects, and stress risers after implant removal make this a technically demanding procedure that is associated with a high incidence of intraoperative and postoperative surgical complications. For instance, intra-operative femur fracture, stem subsidence or loosening, greater trochanter fracture, periprosthetic fracture or dislocation are some of the complications that may occur [3, 6–9]. Currently, primary and revision femur stems with a cementless or cemented techniques have been utilized in the conversion procedure with satisfactory functional outcome and implant survival [8, 10–16]. However, the choice of stem remains controversial. The only comparative study was conducted by Tsai et al., suggesting that cementless, revision stem or cemented, primary stem should be utilized since these procedures had lower complication rates [3].

In this study, we compared two cementless stem types: primary and non-modular revision stem that were used for patients with failed ITFs. Since fracture healing status and bone stock of a damaged metaphysis may be key factors for the choice between cementless primary or revision stem [3, 17, 18], we defined early (≤3 months) and late fixation failures (> 3 months) of ITF according to the interval between index fracture fixation to the conversion hip arthroplasty procedures based on the average bone union time of around 3 months [18, 19]. We hypothesized that the use of primary stem would lead to higher rates of complication and reoperation in patients who had early fixation failure of ITF.

## Materials and methods

**Fig. 1:**
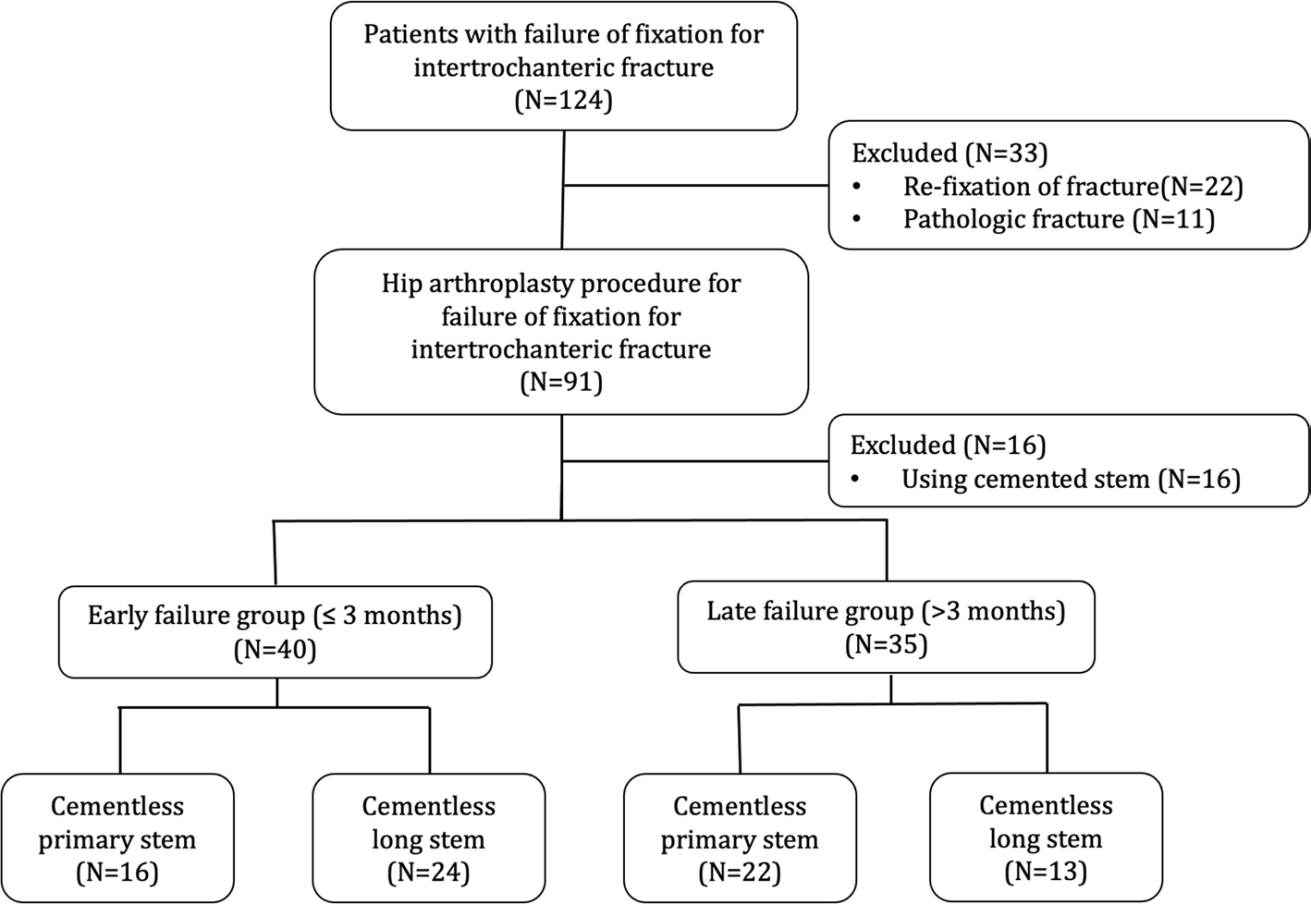
Consort Diagram

In this retrospective cohort study, we included patients that received hip arthroplasty procedures using a cementless primary or revision stem for fixation failure of ITFs (OTA/AO 31-A1, 31-A2 and 31-A3) in a single, tertiary referral center. Traumatology and arthroplasty are two main divisions of the Orthopaedic department in this institute, with approximately 400 hip fracture procedures and 1000 hip arthroplasty procedures being performed per year. This study has been approved by our institutional review board. Our study period was from February 2002 to April 2020. The medical records and pertinent radiographic images from Taipei Veterans General Hospital Orthopaedic database were reviewed. These patients were searched and identified by using Taiwan National Health Insurance procedure codes: “PCS-64170B, PCS-64162B, PCS-64258B, PCS-64201B”, which includes patients that had primary or revision hip arthroplasty procedures during this period. Next, patients that underwent hip arthroplasty procedures for failed treatment of intertrochanteric fractures according to the ICD-10-CM codes: “S72.101G, S72.102G, S72.109G, S72.141-146G, S72.101K, S72.102K, S72.109K, S72.141-146K, S72.21-26XG or S72.21-26XK” were selected for inclusion. The treatment choice for a patient who had failed fixation of ITF was determined based on the condition of femoral head. A revision fixation procedure would be performed in patients who had a preserved femoral head, while a hip arthroplasty procedure would be performed in patients who had a destructed femoral head. Patients who received a revision fixation procedure were excluded (*n* = 22). Cementless femoral stems would be considered first in all hip arthroplasty procedures, except for the severe osteoporotic patients. A cemented stem would then be used. Since a cemented stem was not the first-line treatment option in our institution, we excluded this from our analysis due to the relatively small sample size (*n* = 16). In addition, we excluded pathologic ITFs due to primary or metastatic tumors (*n* = 11). A total of 75 patients (75 hips) fulfilled the search and inclusion criteria. According to the interval from the index fixation procedure to subsequent hip arthroplasty procedure, we stratified these patients into early fixation failure (≤3 months, *n* = 40) and late fixation failure (> 3 months, *n* = 35) (Fig. 1, CONSORT Diagram). Patient demographics are presented in Table 1.

**Table 1:** Patient demographics

Group	Overall (*n* = 75)	Early fixation failure (≤3 months, *n* = 40)	Late fixation failure (> 3 months, *n* = 35)	*P*-value
Stem type				0.040
Primary stem	38 (51%)	16 (40%)	22 (63%)	
Revision stem	37 (49%)	24 (60%)	13 (37%)	
Age (years)	79.3 ± 9.8 (32–96)	80.6 ± 6.2 (67–96)	77.8 ± 12.7 (32–96)	0.241
Sex				0.492
Female	42 (56%)	24 (60%)	18 (51%)	
Male	33 (44%)	16 (40%)	17 (49%)	
Body mass index	24.1 ± 4.7 (16.4–40.3)	23.9 ± 3.9 (18.1–33.7)	24.3 ± 5.4 (16.4–40.3)	0.776
ASA^a^				0.224
1	2 (3%)	0	2 (6%)	
2	45 (60.0%)	23 (57.5%)	22 (63%)	
3	28 (37%)	17 (42.5%)	11 (31%)	
Charlson comorbidity index				0.230
2	3 (4%)	0	3 (9%)	
3	6 (8%)	5 (13%)	1 (3%)	
4	19 (25%)	12 (30%)	7 (20%)	
5+	47 (63%)	23 (57%)	24 (68%)	
Bone mineral density, T-score	−3.1 ± 0.5 (−2.4- -3.8)	−3.0 ± 0.7 (−2.4- -3.8)	−3.1 ± 0.6 (−2.5 - -3.7)	0.523
Interval between index procedure and hip arthroplasty (months)	3 (IQR:9) (1–87)	1(IQR:1) (1–3)	7(IQR:4) (4–87)	< 0.001
Index procedure				
Cephalomedullary nail	24 (32%)	13 (33%)	11 (31%)	1.000
Plate	51 (68%)	27 (67%)	24 (69%)	
Failure of index procedure				0.009
Lag screw cut-out	48 (64%)	31 (78%)	17 (49%)	
Implant breakage or fracture collapse	22 (29%)	9 (22%)	13 (37%)	
Avascular necrosis	5 (7%)	0	5 (14%)	
Hip arthroplasty procedure				
Bipolar hemiarthroplasty	62 (83%)	31 (78%)	31 (89%)	0.206
Total hip arthroplasty	13 (17%)	9 (22%)	4 (11%)	
Surgery duration (mins)	117.4 ± 48.6 (60–300)	118.3 ± 43.1 (60–240)	116.3 ± 54.9 (60–300)	0.862
Intraoperative blood loss (ml)	587.5 ± 399.2 (100–1500)	664.3 ± 298.2 (300–1000)	555.9 ± 438.4 (100–1500)	0.494
Length of stay (days)	7(IQR: 4) (2–94)	8(IQR:6) (2–94)	7(IQR:3) (2–19)	0.090
Follow-up duration (months)	116.2 ± 61.9 (13–234)	120.1 ± 59.4 (13–229)	111.2 ± 65.3 (22–234)	0.481
Mortality rate (one-year mortality)	8 (11%)	5 (13%)	3 (9%)	0.582

^a^None of the patients were in ASA IV ~ VI, *IQR* Interquartile range, The interval between index procedure and hip arthroplasty, and length of stay were expressed as median and IQR

### Surgical techniques and implants

The procedures were performed under general anesthesia, using lateral transgluteal or posterolateral approach. All the procedures were performed in the lateral decubitus position. The femoral head was usually dislocated first before removal of fixation devices. For patients with cut-out of lag screw, we removed the fixation device first and then the femoral head and neck fragments. To perform total hip arthroplasty or bipolar hemiarthroplasty procedure was determined by the surgeon, according to the condition of acetabular cartilage and bony structure. After proper acetabular preparation, a cementless acetabular component was implanted with screw fixation. The femoral canal was opened using a high-speed burr, osteotome or box chisel. A flexible reamer with 2.5-mm ball tipped reaming rod (Synthes, West Chester, PA, USA) with intraoperative fluoroscopy was routinely used for the preparation of femoral canal. After serial reaming and broaching, we inserted the trial stem and checked the size and position using intraoperative fluoroscopy. We then reduced the hip joint, assessed stability and soft tissue tension of the hip. The stem, polyethylene liner and femoral head were then implanted. The greater trochanter fragments along with the attached abductor muscle were reduced and fixed with cerclage wires or nonabsorbable sutures.

**Fig. 2:**
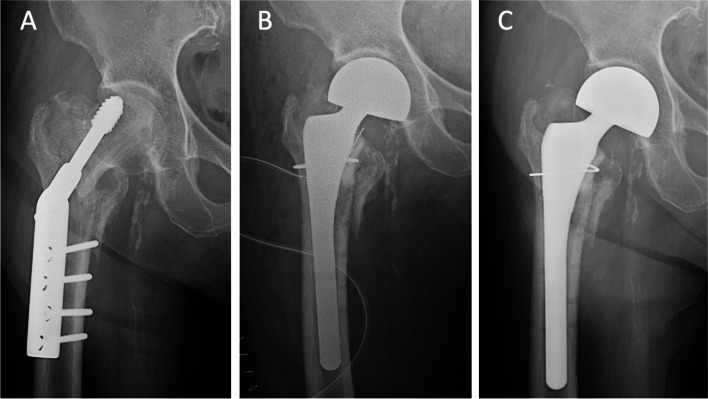
(**A**) 74-year-old female, with early fixation failure (postoperative 6 weeks, fracture collapse) of fixation for intertrochanteric fracture, (**B**) bipolar hemiarthroplasty with cementless primary stem, immediate postoperative radiograph, (**C**) postoperative 36 months

**Fig. 3:**
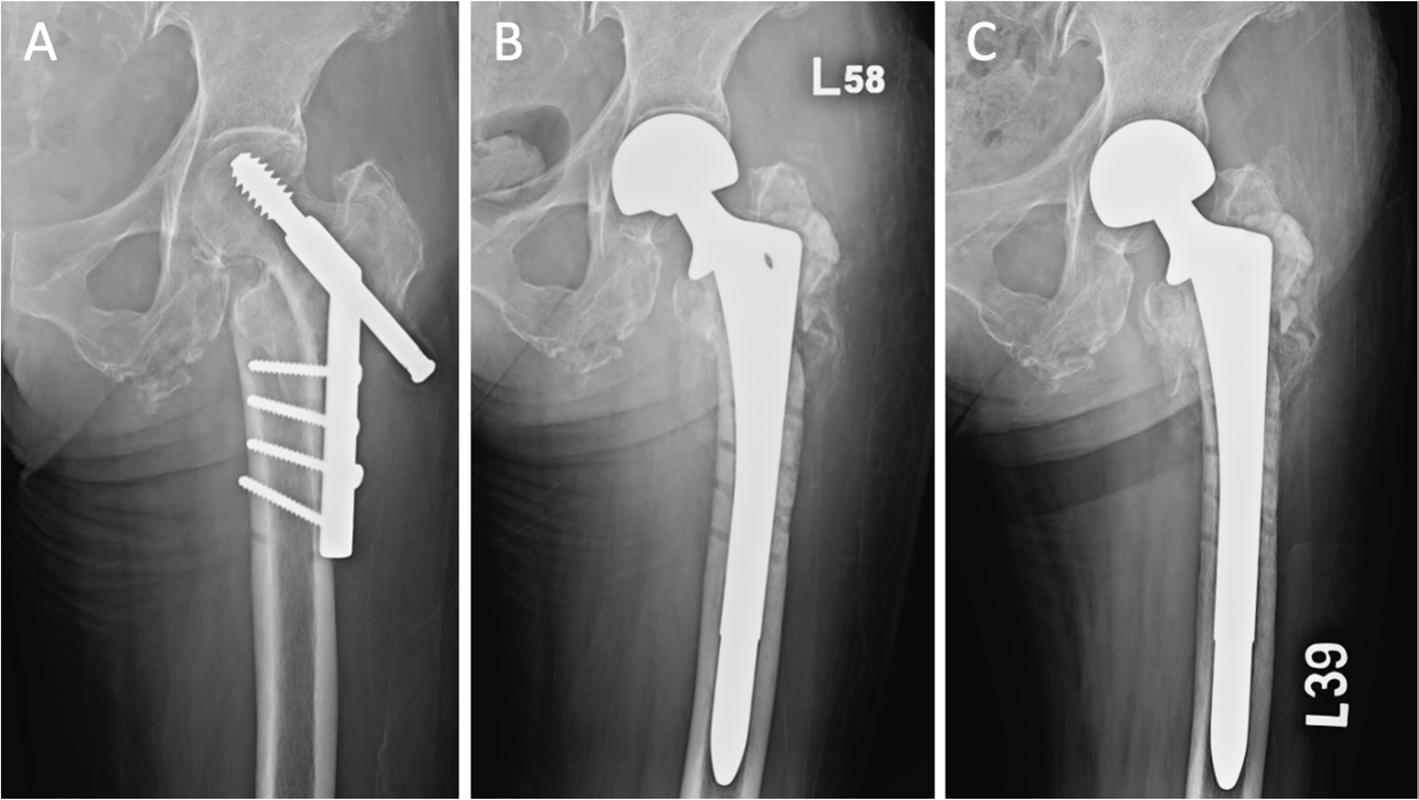
(**A**) 72-year-old female, with early fixation failure (postoperative 4 weeks, fracture collapse), (**B**) bipolar hemiarthroplasty with cementless revision stem, immediate postoperative radiograph, (**C**) postoperative 22 months

The use of a primary or a revision stem during the hip arthroplasty procedure was determined by the surgeon, primarily based on the integrity of the metaphyseal bone stock. A primary stem was utilized in 38 (51%) procedures, while a revision stem was used in the other 37 procedures (49%). In the primary stem cohort (Fig. 2), we included Versys (Zimmer Biomet, Warsaw, IN, USA), M/L taper (Zimmer Biomet, Warsaw, IN, USA), U2 (United, Taiwan) and Secur-fit (Stryker Orthopedics, Mahwah, IN, USA). On the other hand, we included U2 revision (United, Taiwan), Restoration HA (Stryker Orthopedics, Mahwah, IN, USA), AML (Depuy, Warsaw, IN, USA) and Wagner SL (Zimmer Biomet, Warsaw, IN, USA) for the revision stem cohort (Fig. 3).

### Outcome domains

All patients were followed up at postoperative 1 month, 3 months, 6 months and annually thereafter. We recorded peri-operative surgical and medical complications at the follow-up visits. The surgical complications that were recorded included intraoperative femur fracture, stem subsidence or loosening, greater trochanter fracture, periprosthetic fracture, dislocation, periprosthetic joint infection, acetabular wear or cup loosening. The intraoperative femur fracture event was recorded based on the operation note, which was generally defined as fracture that propagated from a pre-existing fracture or a fracture that was not observed on the preoperative plain films. The diagnosis of aseptic loosening was made based on clinical symptoms, presence of radiolucent lines in three or more Gruen zones and/or stem subsidence more than 5 mm on plain radiographs [20–22], intra-operative findings and multiple sets of intra-operative cultures. The medical complications recorded were pneumonia, urinary tract infection, acute coronary syndrome, congestive heart failure, acute kidney injury, gastrointestinal bleeding, deep vein thrombosis, pulmonary embolism and cerebrovascular disease. For patients who underwent fixation procedure for ITF, bone mineral density was evaluated at postoperative 6 weeks during the outpatient visit.

### Statistical analysis

All statistical analyses were performed using SPSS 25.0 (SPSS Inc., Chicago, IL, USA). We presented the data as mean, range, and standard deviation (SD) for continuous variables and as percentages for categorical variables. We determine the normality of distribution of each continuous variable using Kolmogorov-Smirnov test. The student’s *t* test was used to compare differences between the groups for each continuous variable with normal distribution. The Mann-Whitney U rest was used to compare the continuous variables that were not normally distributed. The Chi-square test was used to compare differences between the two groups for each discrete variable. When one or more of the cells in the contingency table had an expected frequency of less than 5, we performed the Fisher’s exact test. A *p*-value < 0.05 was considered statistically significant.

## Results

### Patient demographics

In the early fixation failure group, revision stem was more frequently used (*n* = 24, 60%) while primary stem was more frequently used (*n* = 22, 63%) in the late fixation failure group. The causes of failure were different between the early and late fixation failure groups (*p* = 0.009). The rate of lag screw cut-out was higher in the early fixation failure group (78% vs. 49%), while the rate of implant breakage (37% vs. 22%) and avascular necrosis (14% vs. 0%) were higher in the late fixation failure group. The patient demographics are presented in Table 1.

In Table 2, we presented patient demographics stratified by both interval and choice of stem. In the early fixation failure group, the proportion of male was higher (63%) in the primary stem group while the proportion of female was higher in the revision stem group (75%). Otherwise, patient demographics were not different in patients operated with a primary stem or a revision stem (Table 2).

**Table 2:** Patient demographics stratified by interval and stem type

Group	Early fixation failure (≤3 months, *n* = 40)		*P*-value	Late fixation failure (> 3 months, *n* = 35)		*P*-value
Stem type	Primary stem (*n* = 16)	Revision stem (*n* = 24)		Primary stem (*n* = 22)	Revision stem (*n* = 13)	
Age (years)	81.1 ± 6.6 (67–96)	80.3 ± 6.0 (70–91)	0.946	77.2 ± 14.5 (32–96)	78.8 ± 9.6 (64–94)	0.827
Sex						
Female	6 (37%)	18 (75%)	0.018	10 (45%)	8 (62%)	0.358
Male	10 (63%)	6 (25%)		12 (55%)	5 (38%)	
Body mass index	22.9 ± 3.6 (18.1–30.3)	24.7 ± 4.0 (19.2–33.7)	0.240	24.1 ± 5.7 (16.4–40.3)	24.7 ± 5.0 (18.6–32.7)	0.781
ASA			0.433			0.517
1	0	0		2 (9%)	0	
2	8 (50%)	15 (63%)		13 (59%)	9 (69%)	
3	8 (50%)	9 (37%)		7 (32%)	4 (31%)	
Charlson comorbidity index			0.814			0.545
2	0	0		3 (14%)	0	
3	2 (13%)	3 (13%)		0	1 (8%)	
4	5 (31%)	7 (29%)		3 (14%)	4 (30%)	
5+	9 (56%)	14 (58%)		16 (72%)	8 (62%)	
Bone mineral density, T-score	−3.0 ± 0.9 (−2.4- -4.0)	−3.1 ± 0.8 (−2.6- -3.8)	0.752	−3.1 ± 0.9 (−2.5- -3.7)	−3.0 ± 0.9 (−2.6- -3.7)	0.898
Interval from index procedure and hip arthroplasty (months)	2(IQR:1) (1–3)	1(IQR:1) (1–3)	0.073	12.5(IQR:12) (4–72)	9(IQR:12) (4–87)	0.987
Index procedure			0.177			0.277
Cephalomedullary Nail	3 (19%)	10 (42%)		5 (23%)	6 (46%)	
Plate	13 (81%)	14 (58%)		17 (77%)	7 (54%)	
Surgery duration (mins)	113.9 ± 39.6 (60–180)	121.3 ± 46.0 (60–240)	0689	114.7 ± 43.7 (60–215)	118.8 ± 71.4 (60–300)	0.600
Intraoperative blood loss (ml)	700.0 ± 424.3 (400–1000)	650.0 ± 295.8 (300–1000)	0.898	508.3 ± 426.3 (100–1500)	670.0 ± 495.7 (250–1450)	0.506
Length of stay (days)	8(IQR:3) (4–94)	7.5(IQR:10) (2–83)	0.988	7(IQR: 3) (5–17)	7(IQR:2) (2–19)	0.674
Follow-up duration (months)	132.0 ± 53.1 (53–229)	112.3 ± 63.0 (13–207)	0.304	122.3 ± 67.2 (27–234)	92 ± 59.5 (22–186)	0.234

*IQR* Interquartile range, The interval between index procedure and hip arthroplasty, and length of stay were expressed as median and IQR

### Surgical complications

**Fig. 4:**
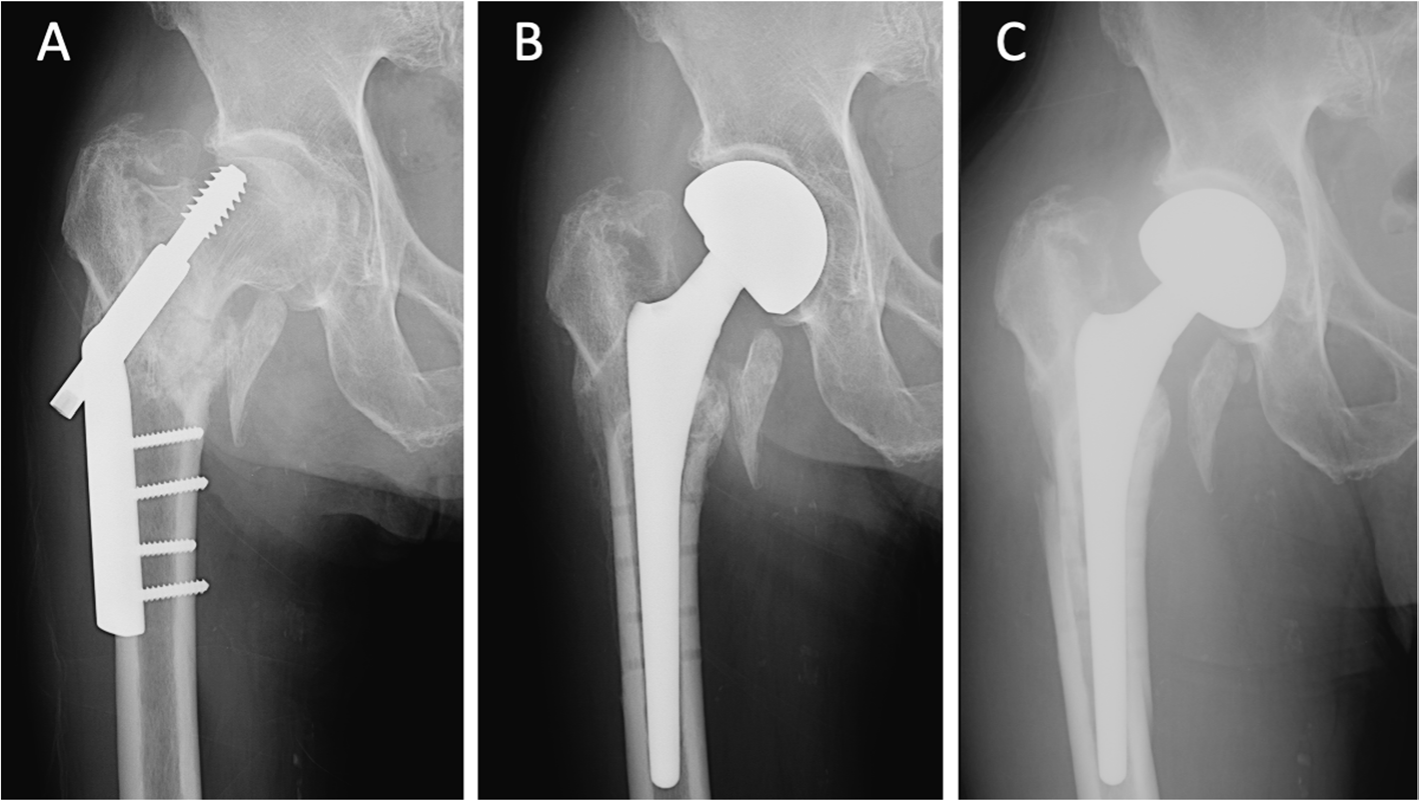
(**A**) 82-year-old male, with early fixation failure (postoperative 6 weeks, fracture collapse), (**B**) bipolar hemiarthroplasty with cementless primary stem, immediate postoperative radiograph, (**C**) periprosthetic femoral fracture at postoperative 4 months

The overall incidence of patients who had surgical complication and reoperation in this cohort was 21% (*n* = 16) and 11% (*n* = 8), respectively. The reason for reoperation included aseptic stem loosening (*n* = 4), periprosthetic fracture without stem loosening (*n* = 2) (Fig. 4), periprosthetic fracture with stem loosening (*n* = 1) and acetabular wear (*n* = 1). The median time from the hip arthroplasty procedure to stem subsidence or loosening were 3 months (interquartile range: 9 months). The proportion of patients with complications were higher in the early fixation failure group than the late fixation failure group (35% vs. 6%, *p* = 0.002).

**Fig. 5:**
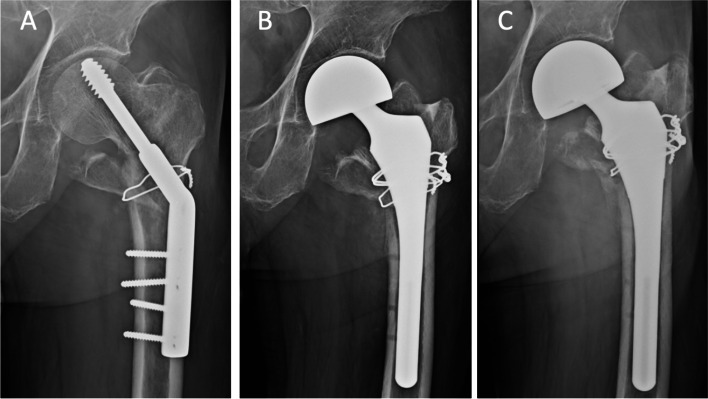
(**A**) 80-year-old male, with early fixation failure (postoperative 8 weeks, fracture collapse), (**B**) bipolar hemiarthroplasty with cementless primary stem, immediate postoperative radiograph, (**C**) stem subsidence at postoperative 6 months

For patients who had early fixation failures of ITF, the use of a primary stem was associated with a higher incidence of stem subsidence or loosening (31% vs. 4%, *p* = 0.019) (Fig. 5) and periprosthetic fracture (19% vs. 0%, *p* = 0.027), compared with the use of a revision stem. On the other hand, there is a trend towards an increased risk of intraoperative fractures with revision stems. Overall, the number of patients who had a surgical complication did not differ between primary or revision stems in patients who had early fixation failure of ITF. However, there was a trend toward a higher reoperation rate (31% vs. 8%, *p* = 0.061) of using primary stems in the early fixation failure group, compared with the revision stem. (Table 3).

**Table 3:** Surgical complications

Group	Overall (*n* = 75)	Early fixation failure (≤3 months, *n* = 40)		*P*-value	Late fixation failure (> 3 months, *n* = 35)		*P*-value
Stem type		Primary stem (*n* = 16)	Revision stem (*n* = 24)		Primary stem (*n* = 22)	Revision stem (*n* = 13)	
Complications (%)							
Intraoperative femur fracture	4 (5%)	0	3 (13%)	0.141	0	1 (8%)	0.187
Stem subsidence or loosening	7 (9%)	5 (31%)	1 (4%)	0.019	0	1 (8%)	0.187
Greater trochanter fracture	5 (7%)	2 (13%)	2 (8%)	0.667	1 (5%)	0	0.435
Periprosthetic fracture	3 (4%)	3 (19%)	0	0.027	0	0	–
Dislocation	1 (1%)	0	0	–	0	1 (8%)	0.187
Periprosthetic joint infection	0	0	0	–	0	0	–
Acetabular complications	1 (1%)	0	1 (4%)	0.408	0	0	–
Number of patients with complications (%)	16 (21%)	7 (44%)	7 (29%)	0.343	1 (5%)	1 (8%)	0.698
Reoperations (%)	8 (11%)	5 (31%)	2 (8%)	0.061	0	1 (8%)	–

Acetabular complications: including acetabular wear or cup loosening

For patients who had late fixation failures of ITF, the number of patients who had a surgical complication and the reoperation rate did not differ between primary or revision stems. (Table 3).

The surgical complication and reoperation rates were similar for patients that received a plate or cephalomedullary nail as the index procedure to treat the ITF (Table S1).

### Medical complications

The incidence of patients who had medical complication in this cohort was 13% (*n* = 10). The incidence was not different between the early and late fixation failure groups. (Table 4).

**Table 4:** Medical complications

Interval from index surgery	Overall (*n* = 75)	Early failure (≤3 months, *n* = 40)	Late failure (> 3 months, *n* = 35)	*P*-value
Pneumonia	7 (9%)	4 (10%)	3 (9%)	0.832
Urinary tract infection	3 (4%)	2 (5%)	1 (3%)	0.637
Acute coronary syndrome	1 (1%)	1 (3%)	0	0.346
Congestive heart failure	1 (1%)	1 (3%)	0	0.346
Acute kidney injury	1 (1%)	1 (3%)	0	0.346
Gastrointestinal bleeding	1 (1%)	1 (3%)	0	0.346
Deep vein thrombosis	0	0	0	–
Pulmonary embolism	0	0	0	–
Cerebrovascular disease	0	0	0	–
Number of patients with complications	10 (13%)	7 (18%)	3 (9%)	0.256

## Discussion

There were two main findings of this study. First, the reason for conversion to hip arthroplasty procedure were different in the early and late fixation failure group. The rate of lag screw cut-out was higher in the early fixation failure group (78% vs. 49%), while implant breakage (37% vs. 23%) and avascular necrosis (14% vs. 0%) were noted more frequently in the late fixation failure group. Second, the use of primary stem was associated with higher rates of stem complications, including stem subsidence or loosening (31% versus 4%) and periprosthetic fracture (19% versus 0%) in the early fixation failure group, compared with the use of revision stem. The rate of surgical complication and reoperation were relatively low, and similar for primary and revision stem in the late fixation failure group.

The distorted anatomy of proximal femur, incompetent abductor mechanism, deficient bone stock, osteoporotic bone quality and stress risers after implant removal are some of the challenges that is encountered during a hip arthroplasty procedure for failed ITFs [6]. As a result, most of the surgical complications (*n* = 20 of 22, 91%) in this study were associated with the femoral component or fractures around proximal femur, including intraoperative femur fracture, stem subsidence or loosening, greater trochanter fracture and periprosthetic fracture. When using a cementless stem during the procedure, the most important goal is to achieve optimal primary stability with adequate bone contact, followed by secondary, biologic osteointegration [23]. It might be questionable whether the bone stock of a damaged metaphysis, usually classified as Paprosky type II femoral defect [17], is appropriate for the use of a primary stem. In our analysis, the use of primary stem was associated with higher risk of stem subsidence or loosening in the early (primary vs. revision: 31% vs. 4%, *p* = 0.019) but not in the late fixation failure group, indicating that the partial or complete bone union around the metaphysis in the late fixation failure group might provide adequate support for the use of a primary stem. On the other hand, an increased risk of intraoperative femur fracture may be attributed to malunion around the meta-diaphysis, eccentric stem implantation due to endosteal sclerotic bone, use of revision stem with large diameter, osteoporotic bone quality, and a mismatch of the implant design and femur anatomy (e.g. coronal and sagittal femoral bowing in the Asian population) [3, 6, 24–27]. Despite the routine use of intraoperative fluoroscopy and flexible reamer during femoral canal preparation and implantation, we still observed a trend toward higher overall risk of intraoperative femur fracture associated with the use of revision stem. Therefore, we believe that there might be a role for the primary stem in certain clinical scenarios such as late fixation failures of ITFs, with the advantages of easier preparation and implantation [3], and possibly lower risk of intraoperative fracture. However, the use of primary stem for early fixation failures of intertrochanteric fracture is not recommended.

The results from a biomechanical study suggested that a 30-mm distance between the most distal residual screw hole and the stem tip might be required to prevent stress concentration [28]. Theoretically, the length of an extensively coated, diaphyseal filling revision stem is adequate to bypass the most distal screw hole to avoid stress concentration. The use of revision stems has been reported to be associated with a low risk of periprosthetic fracture, ranged from 0 to 3.4% [3, 8, 14–16]. Cemented stem might be an effective alternative to fill the defect with cement without the need to bypass the screw holes by the suggested distance [10, 12, 29]. Despite the proposed mechanism and satisfying results from using a revision stem, the use of primary, standard stem still has achieved a success in treating failed intertrochanteric fractures [11, 12]. Lizaur-Utrilla et al. reported 43 patients who had undergone THA procedure using primary stem for healed ITF. The mean interval between the fracture and THA was 64.8 months. All stems had bypassed the screw holes by at least 30 mm. At the mean follow-up of 6.6 years, there were no reported cases of stress fractures. However, two early stem subsidence events (5 mm, 11 mm) were noted, but none of the patients experienced implant failures [11]. Zhang et al. included 19 patients that had undergone hip arthroplasty using a standard femoral stem for failed ITF. The mean interval from fracture to conversion was 40.3 months. Although none of the stems had bypassed the most distal screw holes, none of the patients experienced stress fractures [12]. In our study, the revision stems used had bypassed the most distal screw hole by more than 30 mm, while most of the primary stems (*n* = 32 of 38, 84%) did not extend beyond the most distal screw hole. Interestingly, we observed a higher incidence of periprosthetic fracture of using a primary stem only in the early fixation failure group (primary vs. revision, 19% vs. 0%, *p* = 0.027). There were no periprosthetic fractures of using either stem in the late fixation failure group. These findings might suggest that in the early, failed ITF without solid bony union, inadequate support from the metaphysis and stress concentration from the screw holes might lead to increased risk of periprosthetic fracture. In contrast, the bony union around the metaphysis in the late fixation failure group would be more solid, which provides better support around a primary stem. Therefore, the risk for stress fracture should be comparable to that of a native bone stock, which is compatible to the findings from Zhang et al. [12].

We should recognize some limitations of this study. First, the retrospective design of this study could have led to potential biases, including: 1) patient selection bias; 2) decision of using primary or revision stem based on surgeon’s preference and 3) multiple implant designs of primary and revision stems. Second, based on the limited patient number of this study, it was difficult to detect differences in events such as dislocation, periprosthetic joint infection, acetabular wear, cup loosening or medical complications. Third, we included only two most common stem types in our clinical practice for analysis: cementless primary stem and cementless nonmodular revision stem. Data of other important stem types that were less commonly or have not been used in our practice (e.g., cemented stem or modular primary or revision stem) were not available for analysis. Fourth, with multiple comparisons of the surgical complications, the risk of a false positive result was considerable.

## Conclusions

For early fixation failures of ITFs, we caution against the use of cementless primary stems due to a trend towards an increased risk of reoperations compared to the use of cementless revision stems. However, in late fixation failures of ITFs, there is a role for cementless primary stems.

## Supplementary Information

**Additional file 1: Table S1.** Surgical complications (comparing initial plate fixation and nail fixation).

## Data Availability

The information to access the data used in the study is included within this article.

## References

[1] Kulachote N, Sa-Ngasoongsong P, Wongsak S, Chulsomlee K, Jarungvittayakon C, Fuangfa P, Kawinwonggowit V, Mulpruek P (2019). Correlation between perioperative surgical factors and complications after hip arthroplasty, as a salvage procedure, following failure of internal fixation of osteoporotic intertrochanteric fractures. Orthop Res Rev.

[2] Haidukewych GJ, Berry DJ (2003). Hip arthroplasty for salvage of failed treatment of intertrochanteric hip fractures. J Bone Joint Surg Am.

[3] Tsai SW, Chen CF, Wu PK, Huang CK, Chen WM, Chang MC (2016). Does implant selection impact postoperative complications following hip arthroplasty for failed intertrochanteric fractures? A retrospective comparative study. Artif Organs.

[4] Morice A, Ducellier F, Bizot P, Orthopaedics, Traumatology Society of Western F (2018). Total hip arthroplasty after failed fixation of a proximal femur fracture: analysis of 59 cases of intra- and extra-capsular fractures. Orthop Traumatol Surg Res.

[5] Tsai SW, Lin CJ, Tzeng YH, Lin CC, Huang CK, Chang MC, Chiang CC (2017). Risk factors for cut-out failure of Gamma3 nails in treating unstable intertrochanteric fractures: an analysis of 176 patients. J Chin Med Assoc.

[6] Liu P, Jin D, Zhang C, Gao Y (2020). Revision surgery due to failed internal fixation of intertrochanteric femoral fracture: current state-of-the-art. BMC Musculoskelet Disord.

[7] Haidukewych GJ, Berry DJ (2005). Salvage of failed treatment of hip fractures. J Am Acad Orthop Surg.

[8] Moon NH, Shin WC, Kim JS, Woo SH, Son SM, Suh KT (2019). Cementless total hip arthroplasty following failed internal fixation for femoral neck and intertrochanteric fractures: a comparative study with 3-13 years’ follow-up of 96 consecutive patients. Injury.

[9] Xu Q, Lai J, Zhang F, Xu Y, Zhu F, Lin J, Zhao M, Ye J, Wen L (2019). Poor outcomes for osteoporotic patients undergoing conversion total hip arthroplasty following prior failed dynamic hip screw fixation: a nationwide retrospective cohort study. J Int Med Res.

[10] Morsi EMZ, Drwish AEE, Saber AM, Nassar IM, Zaki AEM (2020). The use of standard cemented femoral stems in Total hip replacement after failed internal fixation of intertrochanteric femoral fractures. J Arthroplast.

[11] Lizaur-Utrilla A, Miralles-Munoz FA, Ruiz-Lozano M, Martinez-Mendez D, Alonso-Montero C, Lopez-Prats FA (2020). Outcomes of Total hip arthroplasty for healed intertrochanteric hip fractures. A matched retrospective cohort study. J Arthroplast.

[12] Zhang B, Chiu KY, Wang M (2004). Hip arthroplasty for failed internal fixation of intertrochanteric fractures. J Arthroplast.

[13] Yu W, Han X, Chen W, Mao S, Zhao M, Zhang X, Han G, Ye J, Chen M, Zhuang J (2020). Conversion from a failed proximal femoral nail anti-rotation to a cemented or uncemented total hip arthroplasty device: a retrospective review of 198 hips with previous intertrochanteric femur fractures. BMC Musculoskelet Disord.

[14] Weiss RJ, Karrholm J, Hailer NP, Beckman MO, Stark A (2012). Salvage of failed trochanteric and subtrochanteric fractures using a distally fixed, modular, uncemented hip revision stem. Acta Orthop.

[15] Shi X, Zhou Z, Yang J, Shen B, Kang P, Pei F (2015). Total hip arthroplasty using non-modular Cementless long-stem distal fixation for salvage of failed internal fixation of intertrochanteric fracture. J Arthroplast.

[16] Thakur RR, Deshmukh AJ, Goyal A, Ranawat AS, Rasquinha VJ, Rodriguez JA (2011). Management of failed trochanteric fracture fixation with cementless modular hip arthroplasty using a distally fixing stem. J Arthroplast.

[17] Valle CJ, Paprosky WG (2003). Classification and an algorithmic approach to the reconstruction of femoral deficiency in revision total hip arthroplasty. J Bone Joint Surg Am.

[18] Lee SR, Kim ST, Yoon MG, Moon MS, Heo JH (2013). The stability score of the intramedullary nailed intertrochanteric fractures: stability of nailed fracture and postoperative patient mobilization. Clin Orthop Surg.

[19] Klima ML (2021). Comparison of early fatigue failure of the TFNa and gamma 3 Cephalomedullary nails in the United States from 2015 to 2019. J Orthop Trauma.

[20] Engh CA, Massin P, Suthers KE (1990). Roentgenographic assessment of the biologic fixation of porous-surfaced femoral components. Clin Orthop Relat Res.

[21] Gruen TA, McNeice GM, Amstutz HC (1979). “Modes of failure” of cemented stem-type femoral components: a radiographic analysis of loosening. Clin Orthop Relat Res.

[22] Engh CA, Hooten JP, Zettl-Schaffer KF, Ghaffarpour M, McGovern TF, Macalino GE, Zicat BA (1994). Porous-coated total hip replacement. Clin Orthop Relat Res.

[23] Kheir MM, Drayer NJ, Chen AF (2020). An update on Cementless femoral fixation in Total hip arthroplasty. J Bone Joint Surg Am.

[24] Abdelaal AH, Yamamoto N, Hayashi K, Takeuchi A, Morsy AF, Miwa S, Kajino Y, Rubio DA, Tsuchiya H (2016). Radiological assessment of the femoral bowing in Japanese population. SICOT J.

[25] Su XY, Zhao Z, Zhao JX, Zhang LC, Long AH, Zhang LH, Tang PF (2015). Three-dimensional analysis of the curvature of the Femoral Canal in 426 Chinese femurs. Biomed Res Int.

[26] Meek RM, Garbuz DS, Masri BA, Greidanus NV, Duncan CP (2004). Intraoperative fracture of the femur in revision total hip arthroplasty with a diaphyseal fitting stem. J Bone Joint Surg Am.

[27] Sumner DR (2015). Long-term implant fixation and stress-shielding in total hip replacement. J Biomech.

[28] Chen DW, Lin CL, Hu CC, Tsai MF, Lee MS (2013). Biomechanical consideration of total hip arthroplasty following failed fixation of femoral intertrochanteric fractures - a finite element analysis. Med Eng Phys.

[29] Enocson A, Mattisson L, Ottosson C, Lapidus LJ (2012). Hip arthroplasty after failed fixation of trochanteric and subtrochanteric fractures. Acta Orthop.

