# Supplementary material for: Is there a role for cementless primary stem in hip arthroplasty for early or late fixation failures of intertrochanteric fractures?
Source: BMC Musculoskelet Disord. 2022 Mar 18;23:266. doi: 10.1186/s12891-022-05223-x (PMC8933997; doi:10.1186/s12891-022-05223-x)
Supplement: Supplementary file 1 — Additional file 1: Table S1. Surgical complications (comparing initial plate fixation and nail fixation). [file 12891_2022_5223_MOESM1_ESM.docx]

Table S1. Surgical complications (comparing initial plate fixation and nail fixation)

| Group | Overall  (n=75) | Plate fixation  (n=51) | Cephalomedullary nail fixation  (n=24) | P-value |
| --- | --- | --- | --- | --- |
| Complications (%) |  |  |  |  |
| Intraoperative femur fracture | 4 (5.3%) | 3(5.9%) | 1(4.2%) | 0.758 |
| Stem subsidence or loosening | 7 (9.3%) | 5(9.8%) | 2(8.3%) | 0.837 |
| Greater trochanter fracture | 5 (6.7%) | 4(7.8%) | 1(4.2%) | 0.552 |
| Periprosthetic fracture | 3 (4.0%) | 3(5.9%) | 0 | 0.547 |
| Dislocation | 1 (1.3%) | 1(2.0%) | 0 | 0.490 |
| Periprosthetic joint infection | 0 | 0 | 0 | - |
| Acetabular complications | 1 (1.3%) | 0 | 1(4.2%) | 0.320 |
| Number of patients with complications (%) | 16 (21.3%) | 11(21.6%) | 5(20.8%) | 0.698 |
| Reoperations (%) | 8 (10.6%) | 5(9.8%) | 3(12.5%) | 0.724 |

Acetabular complications: including acetabular wear or cup loosening
